# Supplementary material for: Live Cell Imaging by Förster Resonance Energy Transfer Fluorescence to Study Trafficking of PLGA Nanoparticles and the Release of a Loaded Peptide in Dendritic Cells
Source: Pharmaceuticals (Basel). 2023 May 31;16(6):818. doi: 10.3390/ph16060818 (PMC10304224; doi:10.3390/ph16060818)
Supplement: Supplementary file 1 [file pharmaceuticals-16-00818-s001.zip › pharmaceuticals-2392201-supplementary.pdf]

pharmaceuticals

MDPI

Supplemental Materials

Live Cell Imaging by Förster Resonance Energy Transfer Fluorescence to Study Trafficking of PLGA Nanoparticles and the Release of a Loaded Peptide in Dendritic Cells

Mengshan Liu [1,2], Chun Yin Jerry Lau [1], Irene Trillo Cabello [1], Johan Garssen [2,3], Linette E. M. Willemsen [2], Wim E. Hennink [1] and Cornelus F. van Nostrum [1,*]

1 Department of Pharmaceutics, Utrecht Institute for Pharmaceutical Sciences, Utrecht University, 3584 CG Utrecht, The Netherlands; m.liu2@uu.nl (M.L.); jerry.lau@bath.edu (C.Y.J.L.); irenichus26@gmail.com (I.T.C.); w.e.hennink@uu.nl (W.E.H.)
2 Department of Pharmacology, Utrecht Institute for Pharmaceutical Sciences, Utrecht University, 3584 CG Utrecht, The Netherlands; j.garssen@uu.nl (J.G.); l.e.m.willemsen@uu.nl (L.E.M.W.)
3 Department of Immunology, Nutricia Research B.V., 3584 CT Utrecht, The Netherlands
* Correspondence: c.f.vannostrum@uu.nl

Calculation and interpretation of FRET efficiency of FRET NP 0.8:1

The actual highest E_{FRET} was calculated from the decrease in the Cy3 fluorescence intensity at $\lambda_{ex/em}$=555/570 nm of a suspension of FRET NP 0.8:1, relative to that of a suspension of Pep-Cy3 NP (with 0.7% loading of Pep-Cy3) at a concentration of 0.5 mg/mL in PBS at room temperature (**equation 1** [1,2]):

$$E_{FRET} = 1 - \frac{\bar{I}_{DA}}{\bar{I}_D}, \quad (1)$$

in which $\bar{I}_{DA}$ and $\bar{I}_D$ are the normalized donor fluorescence (Cy3) intensities at $\lambda_{ex/em}$=555/570 nm in the presence and absence of the acceptor (Cy5), respectively. $\bar{I}_{DA}$ is the donor (Cy3) fluorescence intensity at $\lambda_{ex/em}$=555/570 nm of FRET NP 0.8:1 (1757 A.U.) that was normalized by subtraction of the background signal of 9 wt% PLGA-Cy5 NP (98 A.U.). The donor (Cy3) fluorescence intensity $\bar{I}_D$ (4832 A.U.) was obtained from the 1 wt% Pep-Cy3 NP, however these have a lower loading of Pep-Cy3 (0.3%) than that of the FRET NP 0.8:1 (0.7%). Therefore, we had to correct for this difference and multiplied the measured fluorescence intensity I_D by a factor of 0.7/0.3. These normalizations result finally in the following calculation (**equation 2**):

$$E_{FRET} = 1 - \frac{\bar{I}_{FRET\ NP\ 0.8:1} - \bar{I}_{9\%PLGA-Cy5\ NP}}{\bar{I}_{1\%\ Pep-Cy3\ NP} \times \frac{LC_{FRET\ NP0.8:1}}{LC_{1\%\ Pep-Cy3\ NP}}} = 1 - \frac{1757-98}{4832 \times \frac{0.7}{0.3}} = 0.87\ or\ 87\%, \quad (2)$$

Concluding from this, the encapsulation efficiency of Pep-Cy3 is sufficient to obtain a high FRET efficiency (87%) in the dual labeled FRET NP 0.8:1 dispersed in PBS (pH 7.4). The observed FRET fluorescence is contributed to the large spectral overlap of the selected Cy3-Cy5 pair as donor and acceptor, respectively. The large spectral overlap integral $J(\lambda)$ is calculated from the integral of the normalized donor Cy3 emission intensity $\bar{I}_D (\int \bar{I}_D\, d\lambda = 1)$, multiplied by the extinction coefficient of the acceptor Cy5 ε_A (25,000 $mol^{-1}\ cm^{-1}$) [3] and by the wavelength to fourth power according to the **equation 3**[1].

$$J(\lambda) = \int \bar{I}_D \varepsilon_A \lambda^4\, d\lambda, \quad (3)$$

The observed Förster distance (R_0) is defined as the separation distance between the donor and acceptor that corresponds to 50% FRET efficiency and is calculated with **equation 4** [4].

$$R_0 = 0.0211(\kappa^2 \Phi_D n^{-4} J(\lambda))^{1/6}, \quad (4)$$

Herein, $\kappa^2 = 2/3$ is the FRET orientation factor [5], n = 1.445 is the refractive index of PLGA [6], $\Phi_D = 0.15$ is the donor Cy3 quantum yield [3] and J (λ) (for FRET pair Cy3/Cy5) is calculated as 7.6×10^{15} M^{-1} cm^{-1} nm^4 using equation (1). Thus, R_0 is equal to 50 Å (5.0 nm). Practically, efficient non-radioactive energy transfer only occurs when the separation distance between the acceptor and donor is within the range between 0.5-1.5 R_0 (Förster distance) (2.5-7.4 nm) [3,7]. In conclusion, the occurrence of FRET indicates a proximity (between 2.5-7.4 nm) between the donor and acceptor dyes in the PLGA nanoparticles.

Förster distance (R_0) of 5 nm is the separation distance of D (Cy3) and A (Cy5) in PLGA NP, when assuming a FRET efficiency (E_{FRET}) of 50%. However, the actual E_{FRET} was 87% as calculated by using **equation 3**. Therefore, the actual dye pair distance is smaller. Indeed, the energy transfer mediated by dipole-dipole interactions by the donor and acceptor dye is highly dependent on the separation distance (r), and depends on the Förster distance R_0 as expressed by **equation 5** [1,3]:

$$E_{FRET} = \frac{1}{1+(\frac{r}{R_0})^6}, \quad (5)$$

Therefore, from **equation 1** and the previously calculated E_{FRET} (87%) and R_0 (5.0 nm), the actual average separation distance (r) between the donor (Cy3) and acceptor (Cy5) in the FRET NP 0.8:1 is 3.6 nm, which is again between 0.5-1.5 R_0 (Förster distance, 2.5-7.4 nm) to allow occurrence of FRET signal.

Thus, we can conclude that dual labeled FRET nanocarriers of ~250 nm at a D/A ratio of 0.56:1 enables efficient non-radioactive energy transfer from the Cy3 labeled Peptide to the Cy5 labeled PLGA NP matrix with a close average proximity between Cy3 and Cy5 of 3.6 nm.

Supplemental tables

Supplemental Table S1. Cy3 fluorescence intensity of 1 mg/mL 2% Pep-Cy3 NP in RPMI 1640 medium before and after filtration with 0.2 μm RC membrane syringe filter.

NP suspension	FI[1]-Cy3 at $\lambda_{ex/em}$ = 555/570 nm (A.U.)			
	Non-filtered	Filtered	FI ratio of Filtered/Non-filtered	Filtrated NP[2] (mg/mL)
2% Pep-Cy3 NP	9006	1071	0.12	0.12

[1] FI: Fluorescence intensity

[2] $\text{Concentration}_{\text{filtered NP}}\left(\frac{\text{mg}}{\text{mL}}\right) = \frac{\text{FI}_{\text{filtered NP}}\,(\text{A.U.})}{\text{FI}_{\text{non-filtered NP}}(\text{A.U.})} \times \text{Concentration}_{\text{non-filtered NP}}\left(\frac{\text{mg}}{\text{mL}}\right) = \frac{\text{FI}_{\text{filtered NP}}\,(\text{A.U.})}{\text{FI}_{\text{non-filtered NP}}(\text{A.U.})} \times 1(\frac{\text{mg}}{\text{mL}})$

Supplemental Table S2.[a] Cy3, Cy5 and FRET fluorescence intensity of 0.5 mg/mL 1% Pep-Cy3 NP, 9% PLGA-Cy5 NP and FRET NP 0.8:1 in RPMI 1640 medium before and after filtration with 0.2 μm Regenerated Cellulose membrane syringe filter.

NP suspension	FI‡-Cy3 at $\lambda_{ex/em}$ = 555/570 nm				FI-Cy5 at $\lambda_{ex/em}$ = 646/662 nm				FI-FRET at $\lambda_{ex/em}$ = 555/662 nm				Loaded Pep-Cy3 in the filtration§ (μg/mL)
	FI (A.U.)		FI Ratio of Filtered/Non-filtered	Filtered NP** (mg/mL)	FI (A.U.)		FI Ratio of Filtered/Non-filtered	Filtered NP (mg/mL)	FI (A.U.)		FI Ratio of Filtered/Non-filtered	Filtered NP (mg/mL)	
	Non-filtered	Filtered			Non-filtered	Filtered			Non-filtered	Filtered			
1% Pep-Cy3 NP	1839	1084	0.59	0.30	-	-	-	-	-	-	-	-	0.9
9% PLGA-Cy5 NP	-	-	-	-	10000	1007	0.10	0.05	-	-	-	-	-
FRET NP 0.8:1	2346	620	0.26	0.13	10000	5583	0.56	0.28	2752	750	0.27	0.14	0.9

[a] Cy5 fluorescence intensity of PLGA-Cy5 and FRET NP before filtration exceeded the maximum of 10,000 A.U. that can be measured by our machine. Measured FI-Cy5 of filtered FRET NPs was 5,583 A.U., showing loss of NPs by filtration. Indeed, from Cy3 and FRET fluorescence data (see Table) it can be concluded that just 26-27% of FRET NPs remained after filtration. Therefore, the Cy5 fluorescence intensity of the stock suspension before filtration was expected to have been 5,583/0.265=21,068 A.U. It is assumed that the stock suspension of PLGA-Cy5 NPs would have given the same FI of 21,068 A.U. as for the FRET NPs, since both contained the same amount of Cy5 labeled polymer. After filtration of the PLGA-Cy5 NPs we measured 1007 A.U., which would suggest that only 5% remained after filtration, which is equal to 0.03 mg/mL.

‡ FI: Fluorescence intensity

§ $\text{Concentration}_{\text{loaded Pep-Cy3}} (\frac{\mu g}{mL}) = \text{Concentration}_{\text{filtered NP}} (\frac{mg}{mL}) \times \text{Loading capacity}_{\text{Pep-Cy3}} (\%) \times 1000$

** $\text{Concentration}_{\text{filtered NP}} (\frac{mg}{mL}) = \frac{\text{FI}_{\text{filtered NP}}(\text{A.U.})}{\text{FI}_{\text{non-filtered NP}}(\text{A.U.})} \times \text{Concentration}_{\text{non-filtered NP}} \left(\frac{mg}{mL}\right) = \frac{\text{FI}_{\text{filtered NP}}(\text{A.U.})}{\text{FI}_{\text{non-filtered NP}}(\text{A.U.})} \times 0.5 \left(\frac{mg}{mL}\right)$

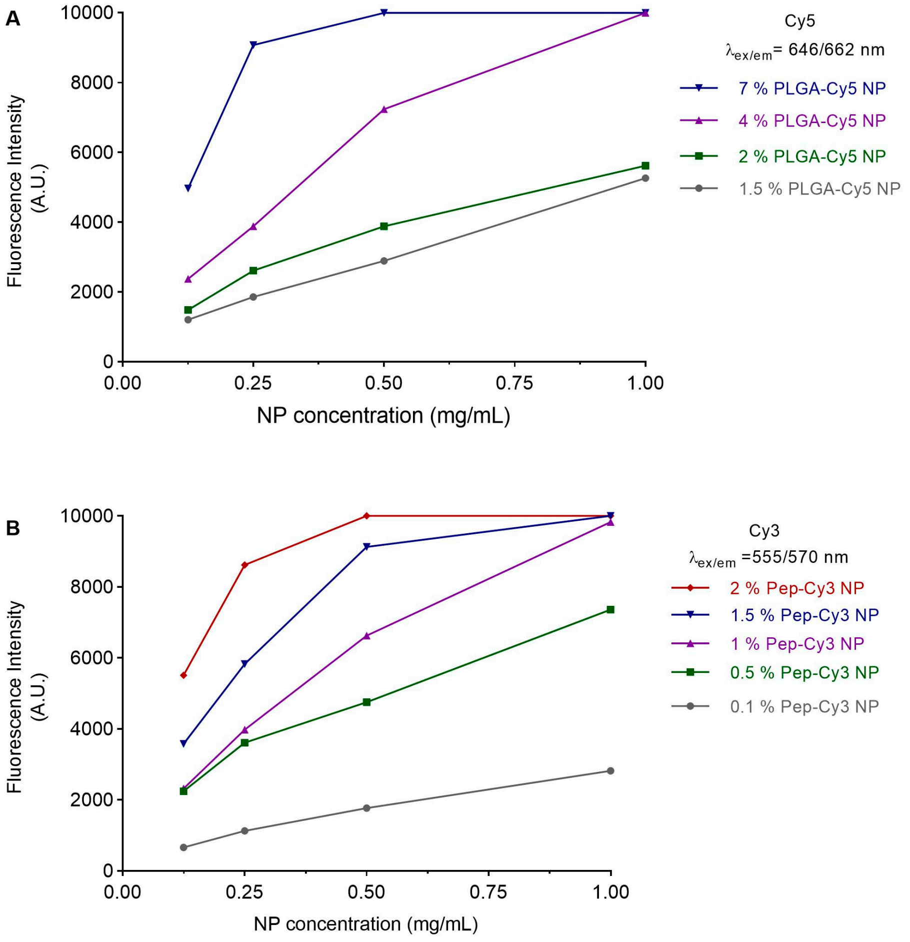

Supplemental Figure S1. Concentration dependent fluorescence intensity of (A) 1.5-7% PLGA-Cy5 NPs at $\lambda_{ex/em}$=646/662 nm and 0.1-2% Pep-Cy3 NPs $\lambda_{ex/em}$=555/570 nm in PBS.

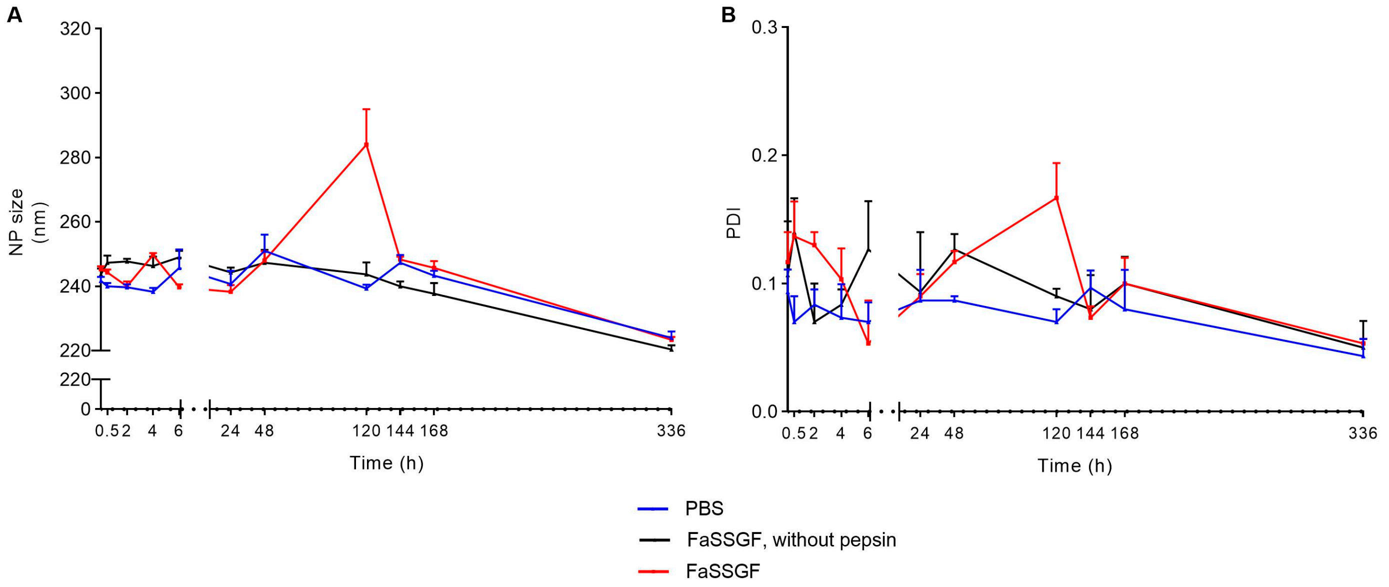

Supplemental Figure S2. Mean NP size (A) and polydispersity index (PDI) (B) of empty PLGA NP incubated in different media at 37 °C over 336 h as determined by Zetasizer Nano S. Data are presented as mean±SEM, and SEM is derived from 3 different measurements of one sample.

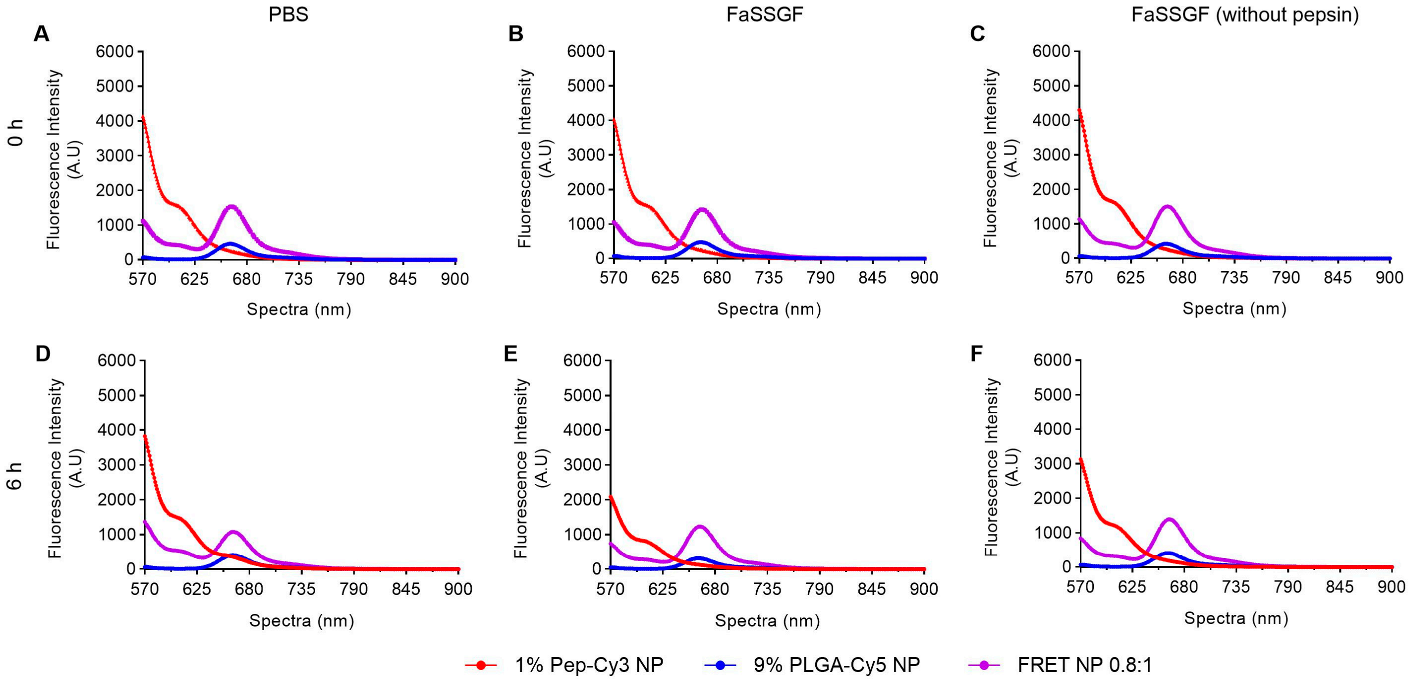

Supplemental Figure S3. Fluorescence spectra obtained after 0 h (top) and 6 h (bottom) at $\lambda_{ex/em}$=555/570-900 nm of 0.2 mg/mL 1% Pep-Cy3 NP, 9% PLGA-Cy5 NP and FRET NP 0.8:1 (1% Pep-Cy3:9% PLGA-Cy5) incubated at 37 °C in (A) PBS (pH 7.4), (B) in FaSSGF (pH 1.6) with pepsin and (C) in FaSSGF (pH 1.6) without pepsin for 0 and 6 h.

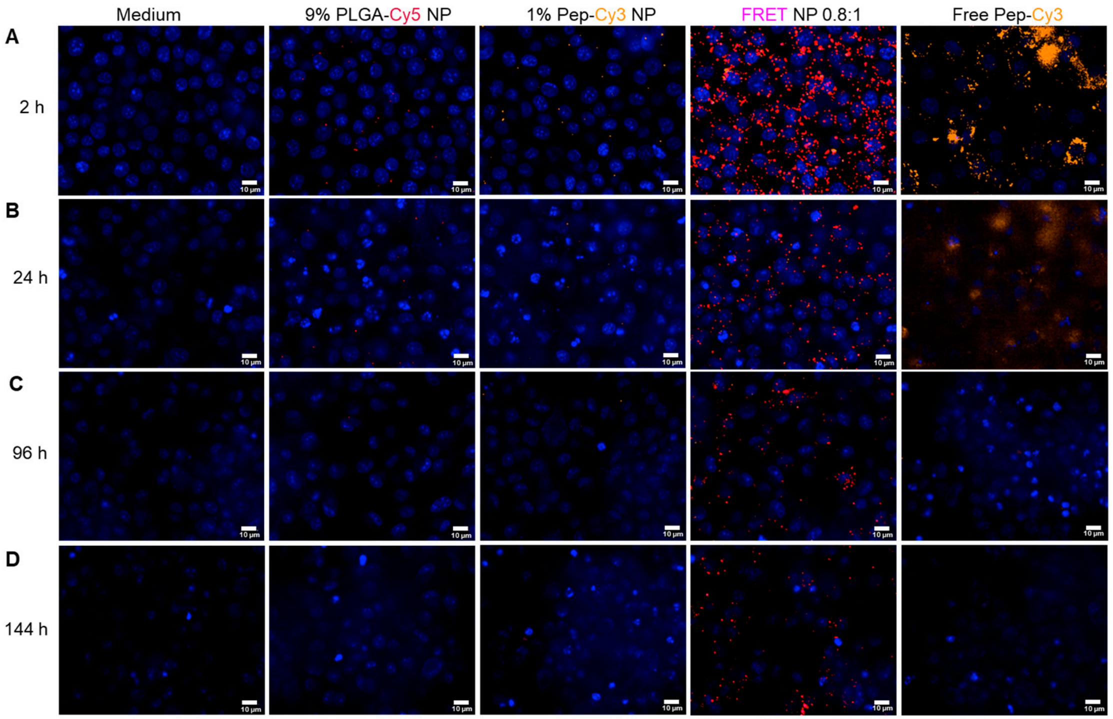

Supplemental Figure S4. Cellular images of internalized fluorescent nanoparticles and free Pep-Cy3 by murine DC 2.4. According to the scheme of Figure 8A, cells were washed and live imaged over 144 h after 2.5 h-incubation of DC 2.4 cells with medium, 9% PLGA-Cy5 NP, 1% Pep-Cy3 NP, FRET NP 0.8:1 (1% Pep-Cy3:9% PLGA-Cy5) or 1 µg/mL free Pep-Cy3. Shown are representative merged confocal fluorescence microscopy images (B) of nuclei (Hoechst, blue, λ_{ex}=405±5 nm), Cy3 (orange, λ_{ex}=561±2 nm), Cy5 (red, λ_{ex}=640+4/-5 nm) and FRET (λ_{ex}=561±2 nm) taken at (A) 2, (B) 24, (C) 96 and (D) 144 h after the washing step, bars indicate 10 µm.

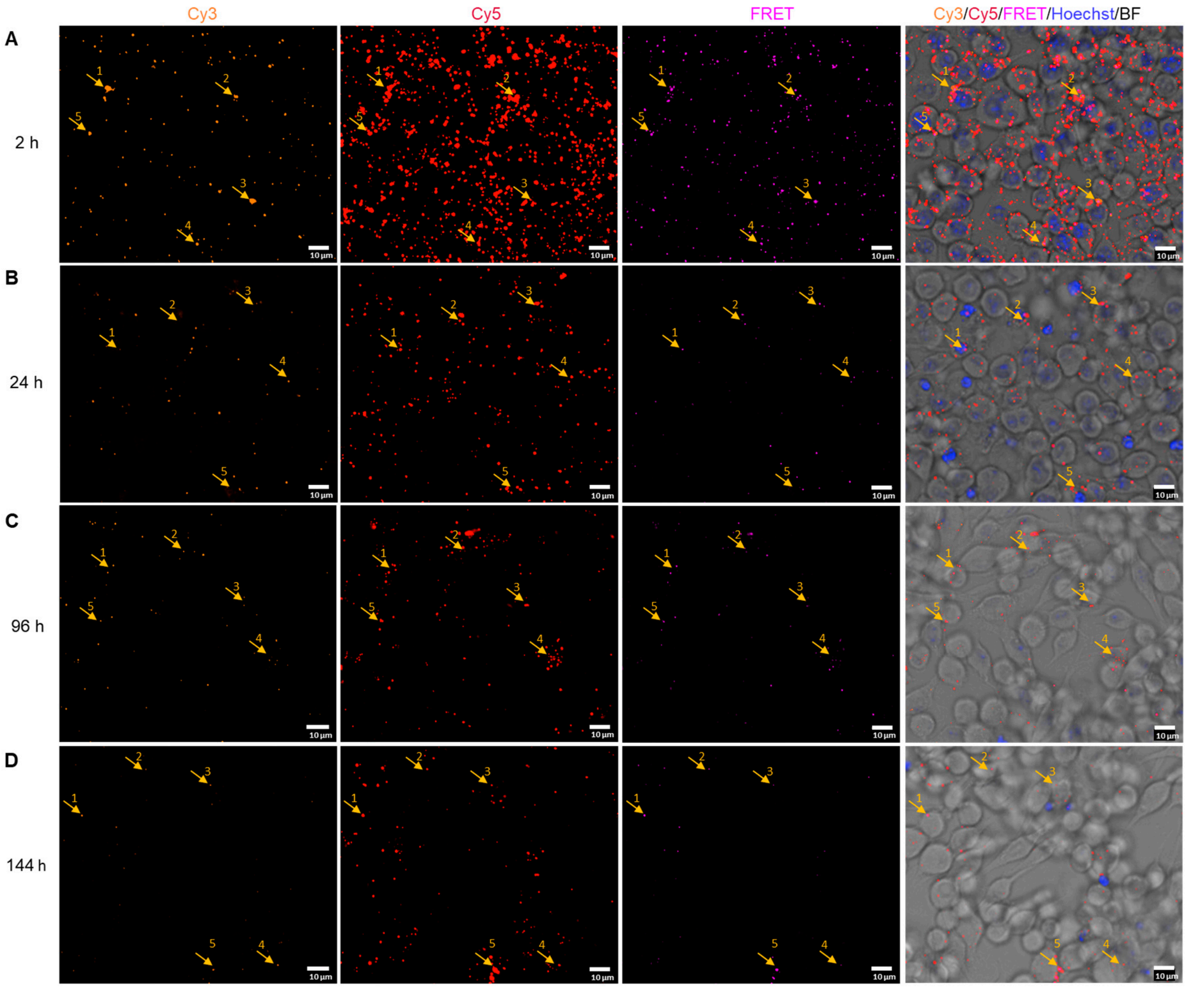

Supplemental Figure S5. Intracellular localization of FRET NP after internalization by DC 2.4 cells. According to the scheme of Figure 8A, DC 2.4 cells were incubated with FRET NP 0.8:1 (1% Pep-Cy3:9% PLGA-Cy5) for 2.5 h and subsequently washed. Shown are representative confocal fluorescence microscopy images of DC 2.4 cultured with FRET NP at (A) 2, (B) 24, (C) 96 and (D) 144 h after the washing step respectively, bars indicate 10 µm. Colocalizations of Cy3 (orange, λ_{ex}=561±2 nm), Cy5 (red, λ_{ex}=640+4/-5 nm) and FRET (purple, λ_{ex}=561±2 nm) fluorescence are indicated with the yellow arrows and numbers.

References

1. Algar, W.R.; Hildebrandt, N.; Vogel, S.S.; Medintz, I.L. FRET as a biomolecular research tool - understanding its potential while avoiding pitfalls. *Nat Methods* **2019**, *16*, 815-829, doi:10.1038/s41592-019-0530-8.
2. Sapsford, K.E.; Berti, L.; Medintz, I.L. Materials for fluorescence resonance energy transfer analysis: beyond traditional donor-acceptor combinations. *Angew Chem Int Ed Engl* **2006**, *45*, 4562-4589, doi:10.1002/anie.200503873.
3. Ishikawa-Ankerhold, H.C.; Ankerhold, R.; Drummen, G.P. Advanced fluorescence microscopy techniques--FRAP, FLIP, FLAP, FRET and FLIM. *Molecules* **2012**, *17*, 4047-4132, doi:10.3390/molecules17044047.
4. Swider, E.; Maharjan, S.; Houkes, K.; van Riessen, N.K.; Figdor, C.; Srinivas, M.; Tagit, O. Forster Resonance Energy Transfer-Based Stability Assessment of PLGA Nanoparticles in Vitro and in Vivo. *ACS Appl Bio Mater* **2019**, *2*, 1131-1140, doi:10.1021/acsabm.8b00754.
5. Joseph V. Mersol, H.W., Ari Gafni, and Duncan G. Steel. Consideration of dipole orientation angles yields accurate rate equations for energy transfer in the rapid diffusion limit. *Biophys. J.* **1992**, *61*, 1647-1655.
6. Ismail, R.; Sovany, T.; Gacsi, A.; Ambrus, R.; Katona, G.; Imre, N.; Csoka, I. Synthesis and Statistical Optimization of Poly (Lactic-Co-Glycolic Acid) Nanoparticles Encapsulating GLP1 Analog Designed for Oral Delivery. *Pharm Res* **2019**, *36*, 99, doi:10.1007/s11095-019-2620-9.
7. Förster, V.T. Zwischenmolekulare Energiewanderung und Fluoreszenz. *Annalen der Physik.* **1948**, *6*, 9.
